# A Rare Case of Myasthenia Gravis With Underlying Aldolase A Deficiency: Diagnostic and Therapeutic Challenges

**DOI:** 10.7759/cureus.86244

**Published:** 2025-06-17

**Authors:** Niyas Khalid Ottu Para, Divyashri Ramanathan Nagarajan, Daya Mani Jacob, Diya Viju

**Affiliations:** 1 Internal Medicine, Burjeel Medical City, Abu Dhabi, ARE

**Keywords:** acetylcholine receptor antibody, aldolase deficiency, autoimmune neuromuscular junction disorder, metabolic myopathy, myasthenia gravis, recurrent rhabdomyolysis

## Abstract

We report one of the first documented cases globally of a 24-year-old female presenting with recurrent episodes of muscle pain, weakness, and rhabdomyolysis. Although she initially improved with conservative management, her symptoms recurred, warranting further investigation. Comprehensive serologic and metabolic evaluations revealed a rare dual diagnosis of seropositive myasthenia gravis (MG) and aldolase A deficiency. MG, an autoimmune neuromuscular junction disorder, contributed to her characteristic diurnal fatigue and acetylcholine receptor antibody positivity, while aldolase A deficiency, a rare metabolic myopathy, was responsible for impaired glycolysis and exertional rhabdomyolysis. This case underscores the importance of considering overlapping autoimmune and metabolic neuromuscular conditions in young adults with unexplained muscle breakdown.

## Introduction

Myasthenia gravis (MG) is an autoimmune neuromuscular disorder marked by fluctuating skeletal muscle weakness due to antibodies targeting components of the neuromuscular junction, most commonly the acetylcholine receptor (AChR). While prevalence is similar between sexes, MG tends to appear earlier in females and later in males. Ptosis, diplopia, bulbar weakness, and limb fatigue are common symptoms. Diagnosis is supported by autoantibody testing against AChR, muscle-specific kinase (MuSK), or low-density lipoprotein receptor-related protein 4 (LRP4), and by electrophysiological studies [1]. The disease exhibits a bimodal age distribution, with early-onset MG more prevalent in females and late-onset MG more common in males. Approximately 10%-20% of patients with AChR-positive MG have an associated thymoma [2]. Treatment includes symptomatic management, immunosuppressants, and in some cases, thymectomy. Recent advances have introduced targeted therapies aimed at reducing corticosteroid dependence and improving outcomes in refractory cases [3].

Aldolase deficiency is a rare, autosomal recessive metabolic disorder affecting glycolysis, particularly in skeletal muscle. Aldolase A catalyzes the reversible conversion of fructose-1,6-bisphosphate to glyceraldehyde-3-phosphate and dihydroxyacetone phosphate. As observed in this patient, its deficiency impairs adenosine triphosphate (ATP) generation during anaerobic exertion, leading to exercise-induced muscle breakdown, rhabdomyolysis, and myoglobinuria [4]. Symptoms often follow strenuous activity, but in some cases, as in this report, episodes may recur with minimal exertion, suggesting progressive metabolic vulnerability of skeletal muscle [5].

To our knowledge, this is the first reported case describing the coexistence of an autoimmune neuromuscular disorder like MG and a metabolic myopathy such as aldolase A deficiency. While the co-occurrence may be coincidental, it prompts speculation about a possible underlying pathophysiologic connection. Emerging evidence suggests that metabolic stress within skeletal muscle may contribute to immune dysregulation and autoantigen presentation, potentially triggering or exacerbating autoimmune responses in genetically predisposed individuals [6]. For instance, alterations in glucose metabolism have been shown to affect the function and differentiation of various immune cells, including T cells and B cells, which are central to the pathogenesis of MG. Moreover, mitochondrial dysfunction, a hallmark of several metabolic disorders, has been implicated in the dysregulation of innate and adaptive immunity, contributing to the development of autoimmune diseases [6,7].

Conversely, chronic immune activation and antibody-mediated impairment of neuromuscular transmission in MG may lead to secondary metabolic strain and muscle fiber vulnerability, unmasking latent metabolic deficiencies. This overlap poses significant diagnostic and therapeutic challenges, particularly when symptoms of fatigue, muscle breakdown, and weakness persist despite standard immunomodulatory treatment. By documenting this unique dual pathology, our case underscores the importance of considering both metabolic and autoimmune mechanisms in patients presenting with atypical or refractory neuromuscular symptoms.

## Case presentation

A 24-year-old previously healthy female presented with bilateral leg pain, muscle cramping, generalized muscle weakness, and dark-colored urine following a moderately intense gym workout. Initial laboratory evaluation revealed markedly elevated creatine phosphokinase (CPK) and myoglobin, consistent with rhabdomyolysis. She was treated conservatively with intravenous fluids and rest, which led to temporary symptom resolution.

**Figure 1:**
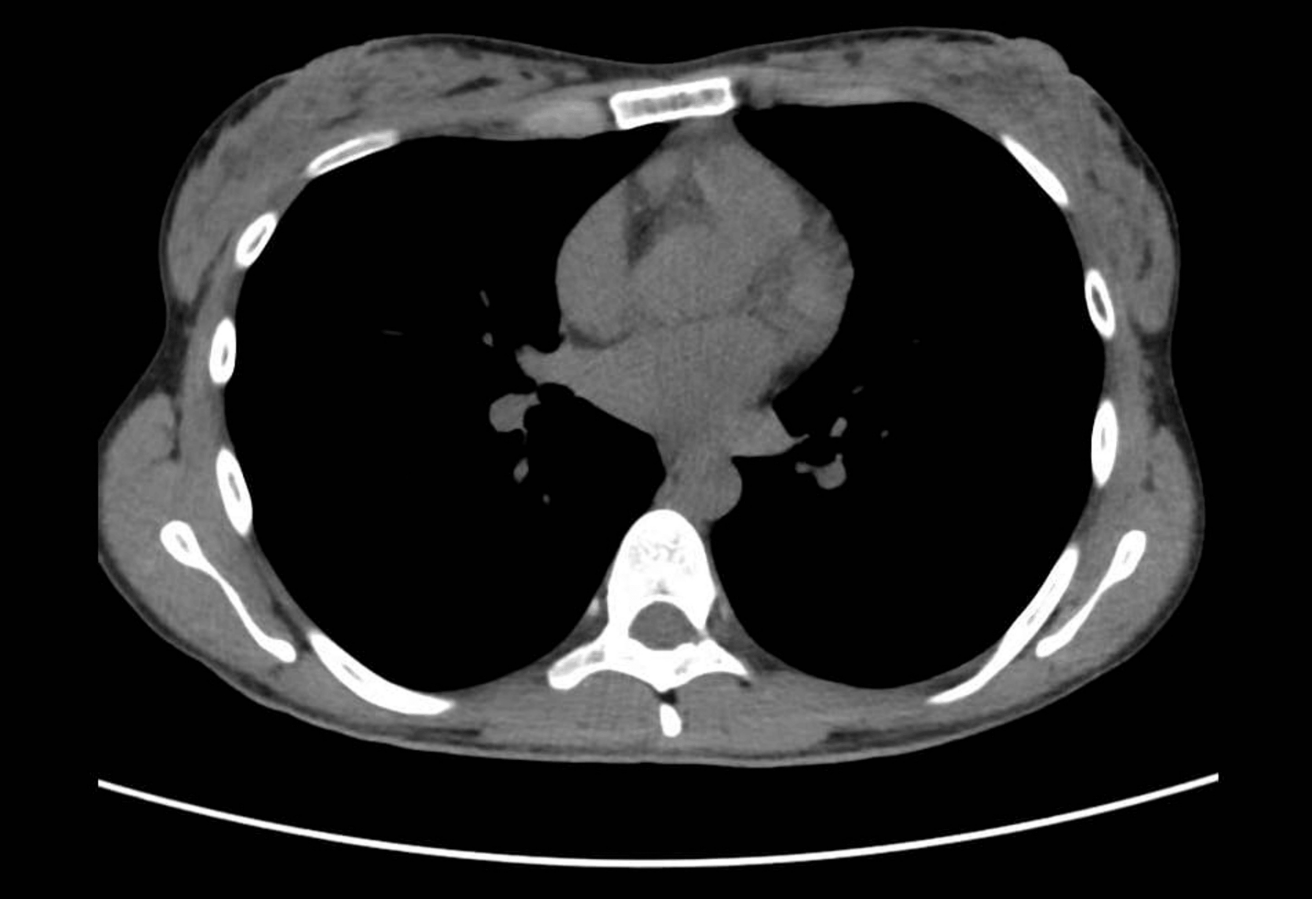
CT chest showing no evidence of mediastinal mass or thymoma.

However, a few weeks later, the patient returned with similar complaints of muscle pain, fatigue, and dark urine despite lifestyle modifications and avoidance of strenuous activity. A detailed clinical history revealed multiple similar episodes since April 2023, each followed by a prolonged recovery period. Fatigue was noted to have a diurnal pattern, worsening throughout the day and improving with rest. She denied any prior history of chronic illness, including diabetes, hypertension, or cardiovascular disease. Her serum CPK was markedly elevated on re-evaluation (Table 1). Further serologic and neurologic investigations were conducted to evaluate for neuromuscular and autoimmune causes. Neurological examination revealed mild bilateral ptosis, more pronounced in the evening, with normal pupillary responses and no diplopia. There was no facial or bulbar weakness. Limb strength was preserved but showed fatigability with sustained activity. Deep tendon reflexes were normal. The acetylcholine receptor (AChR) antibody was positive, suggesting a neuromuscular junction disorder, while anti-MuSK antibodies were negative. Nerve conduction studies showed normal peripheral nerve function, and repetitive nerve stimulation (RNS) did not demonstrate a decremental response. A chest CT scan showed no evidence of thymoma or mediastinal mass (Figure 1). An extractable nuclear antigen (ENA) panel, including anti-Mi-2, anti-Ku, anti-PM-Scl-70, and anti-histone antibodies, was negative, ruling out overlapping autoimmune myopathies. Her aldolase level was significantly reduced, consistent with aldolase A deficiency, a rare metabolic myopathy. Genetic testing was advised but not conducted due to insurance constraints.

**Table 1:** Serial laboratory investigations. *ENA panel includes anti-Mi-2, anti-Ku, anti-PM-Scl-70, and anti-histone antibodies. This table summarizes key laboratory findings from three different time points during the patient’s clinical course. The initial presentation was marked by severe rhabdomyolysis, as evidenced by elevated CPK and myoglobin levels. On follow-up, rhabdomyolysis recurred despite reduced physical activity. Persistently elevated AChR antibody titers, confirmed through both local and international evaluations, supported the diagnosis of seropositive myasthenia gravis. Aldolase levels were low, suggesting aldolase A deficiency. The autoimmune panel ruled out overlapping inflammatory myopathies. CPK: Creatine Phosphokinase; ENA Panel: Extractable Nuclear Antigen Panel; AChR: Acetylcholine Receptor; Anti-MuSK: Anti-Muscle-Specific Tyrosine Kinase.

Parameter	Unit	Reference Range	Initial Presentation	Follow-up Visit	Second Evaluation (Thailand)
Creatine Phosphokinase (CPK)	U/L	0-170	91,632	88,583	91,000
Myoglobin	µg/L	19-51	10,937	Not tested	Not tested
AChR Antibody	nmol/L	>0.50 (positive)	0.88	Not repeated	0.97
Anti-MuSK Antibody	—	Negative	Negative	Not repeated	Not repeated
Aldolase	U/L	3-8	1.2	1	Not tested
ENA Panel*	—	Negative	Negative	Not repeated	Not repeated

The diagnosis of seropositive generalized MG was confirmed based on her characteristic diurnal fatigue, persistently elevated AChR antibody titers, and the exclusion of alternative neuromuscular and autoimmune conditions. Although RNS findings were normal, this does not exclude MG, particularly when supported by positive serology and compatible clinical features. The diagnosis was further substantiated when the patient sought a second opinion in Thailand and underwent extensive evaluation, where repeat testing confirmed AChR antibody positivity. Dietary modifications were recommended for aldolase deficiency, including the restriction of fructose, sucrose, sorbitol, and other lifestyle adjustments. Additionally, reduced physical activity was advised, and the patient was encouraged to increase fluid intake.

The patient was started on pyridostigmine 60 mg three times daily for symptomatic relief and prednisolone 10 mg daily for immunomodulation. She also began monthly intravenous immunoglobulin (IVIG) therapy for MG. A total of 1200 ml of 10% IVIG was administered over three days (400 ml/day). The infusion was titrated at increasing rates: 15 ml/hour for 30 minutes, 30 ml/hour for the next 30 minutes, and 45 ml/hour thereafter. She continued on pyridostigmine and showed clinical improvement with treatment. She did not tolerate oral prednisolone but improved on pyridostigmine.

## Discussion

This case highlights a rare and clinically significant coexistence of aldolase A deficiency, a metabolic myopathy, and seropositive MG in a young woman with recurrent rhabdomyolysis and diurnal fatigue. The presence of these distinct pathologies underscores the intricate relationship between autoimmune dysfunction and energy metabolism in neuromuscular disorders. Aldolase A deficiency is an autosomal recessive disorder that disrupts glycolysis in skeletal muscle, impairing ATP production during exertion and resulting in exercise-induced rhabdomyolysis, myoglobinuria, and muscle cramping [3,4].

Although episodes are typically triggered by intense physical activity, the recurrence of symptoms with minimal exertion in this patient suggests a progressive vulnerability of skeletal muscle energy metabolism. However, aldolase deficiency alone does not fully explain the patient’s diurnal fatigue, a hallmark of MG, nor her positive AChR antibodies. Despite a normal RNS test and nerve conduction studies, the serological findings and characteristic fatigue pattern supported a diagnosis of seropositive MG, which may be present even in the absence of electrophysiological abnormalities, particularly early in the disease course or when the appropriate muscles are not tested [1,8].

The clinical overlap between these two disorders is striking. Both can present with proximal muscle weakness, fatigue, and reduced exercise tolerance. Yet, their underlying pathophysiology differs: MG arises from autoantibody-mediated disruption of neuromuscular transmission, whereas aldolase deficiency results from intrinsic energy failure within the myocyte [2,5]. This shared symptomatology can obscure diagnosis and delay targeted treatment. Notably, rhabdomyolysis is not a typical feature of MG, and its presence should prompt evaluation for coexisting metabolic myopathies, as demonstrated in this case.

Interestingly, recent studies have identified anti-aldolase A antibodies in subsets of seronegative MG patients, suggesting a potential autoimmune response against metabolic enzymes in some neuromuscular conditions [9]. While the patient’s aldolase deficiency appears to be primary, based on significantly reduced enzyme activity (<1.2 U/L), its coexistence with MG raises the possibility that chronic metabolic stress may contribute to or exacerbate immune responses at the neuromuscular junction. The negative RNS, normal nerve conduction studies, and negative autoimmune myopathy panel complicated the diagnostic picture. However, persistently elevated CPK, repeated AChR antibody positivity, and a classic fatigue pattern provided sufficient evidence to support the diagnosis of MG (Table 2) [1,4].

**Table 2:** Comparison between myasthenia gravis and aldolase A deficiency. AChR: Acetylcholine receptor antibody; MuSK: Muscle-specific kinase antibody.

Feature	Myasthenia Gravis (MG)	Aldolase A Deficiency	Similarities
Etiology	Autoimmune neuromuscular junction disorder	Inherited metabolic myopathy (enzyme deficiency)	Both affect muscle function
Pathophysiology	Autoantibodies against AChR or MuSK impair neuromuscular transmission	Deficiency of aldolase A enzyme impairs glycolysis in skeletal muscles	Can present with fatigue and muscle weakness
Onset	Variable; often in young adults or elderly	Typically in childhood or adolescence	Symptoms may be triggered by exertion or stress
Key Symptoms	Ptosis, diplopia, bulbar weakness, limb weakness	Exercise-induced muscle pain, rhabdomyolysis, dark-colored urine	Muscle symptoms triggered by activity
Diagnostic Tests	AChR/MuSK antibodies, edrophonium test	Genetic testing, aldolase assay, muscle biopsy	Require serological and specialized diagnostic tests
Treatment	Acetylcholinesterase inhibitors, immunosuppressants	Supportive care, trigger avoidance	Require lifestyle modifications

Management required a multifaceted approach. The patient responded to pyridostigmine for symptomatic relief, while low-dose corticosteroids and monthly IVIG were initiated for immunomodulation of MG [10]. The IVIG infusion protocol was titrated to minimize the risk of infusion-related reactions. As no enzyme replacement therapy exists for aldolase A deficiency, conservative management remains the standard of care and includes activity restriction, adequate hydration, dietary modifications (including restriction of fructose, sucrose, and sorbitol), and monitoring for renal complications [11].

Traditional broad-based immunotherapies such as corticosteroids, azathioprine, mycophenolate, tacrolimus, and cyclosporine have shown effectiveness in MG but are often limited by delayed onset of action and long-term adverse effects. Recent advances in immunology and pharmacotherapy have introduced novel targeted treatments that directly address the underlying immune dysfunction in MG. These include complement inhibitors (eculizumab, zilucoplan, ravulizumab), Fc receptor inhibitors (efgartigimod, nipocalimab), B-cell depleting agents (targeting CD19, CD20, and BAFF), proteasome inhibitors, T-cell and cytokine-directed therapies (including CAR-T cells), autologous stem cell transplantation, and subcutaneous immunoglobulin (SCIG). Compared to conventional therapies, these emerging treatments offer a more rapid onset of action, improved safety profiles, and the potential for sustained remission [12].

The presence of underlying aldolase A deficiency in a patient with MG may have contributed to recurrent episodes of rhabdomyolysis, even with relatively mild exertion, highlighting the need for deeper exploration into the overlapping and distinct pathophysiological mechanisms of both conditions. The amplified clinical presentation resulting from the coexistence of two rare and unrelated disorders underscores the importance of a thorough diagnostic workup. A comprehensive evaluation, including metabolic, autoimmune, and electrophysiological assessments, is particularly essential in young patients presenting with recurrent muscle symptoms and a suboptimal response to standard therapies.

## Conclusions

In young adults presenting with recurrent rhabdomyolysis and progressive fatigue, clinicians should consider the possibility of overlapping metabolic and autoimmune neuromuscular disorders. The rare coexistence of MG and aldolase A deficiency has important diagnostic and therapeutic implications, as the underlying metabolic defect may exacerbate symptoms even with minimal exertion. The amplified clinical presentation in such cases underscores the need for early recognition, comprehensive evaluation of the underlying pathophysiology, and a multidisciplinary approach to management. Understanding the interaction between these two rare conditions is essential for preventing complications and formulating individualized treatment strategies.
